# Implementation of continuous quality improvement in Aboriginal and Torres Strait Islander primary health care in Australia: a scoping systematic review

**DOI:** 10.1186/s12913-018-3308-2

**Published:** 2018-07-11

**Authors:** Karen Gardner, Beverly Sibthorpe, Mier Chan, Ginny Sargent, Michelle Dowden, Daniel McAullay

**Affiliations:** 1 Public Service Research Group, Business School, UNSW Canberra, Canberra, Australia; 2 SenseMakers 4 Smarter Care, Port Macquarie, NSW 2444 Australia; 3 Australian Primary Health Care Research Institute, Australian National University, Canberra, ACT 0200 Australia; 4 Research, Evaluation and Public Health Nutrition Section, Population Health Division, Health Improvement Branch, ACT Health, Canberra, ACT 260 Australia; 5 One Disease, Menzies Building, RDH Campus, Rocklands Drive, Tiwi, NT 0810 Australia; 6 Kurongkurl Katitjin, Edith Cowan University, 2 Bradford St, Mount Lawley, WA 6050 Australia

**Keywords:** Continuous quality improvement, CQI, Primary health care, Indigenous health, Quality, Barriers and enablers

## Abstract

**Background:**

Continuous Quality Improvement (CQI) programs have been taken up widely by Indigenous primary health care (PHC) services in Australia and there has been national policy commitment to support this. However, international evidence shows that implementing CQI is challenging, impacts are variable and little is known about the factors that impede or enhance effectiveness. A scoping review was undertaken to explore uptake and implementation in Indigenous PHC, including barriers and enablers to embedding CQI in routine practice. We provide guidance on how research and evaluation might be intensified to support implementation.

**Methods:**

Searches were conducted in MEDLINE, CINAHL and the Cochrane Database of Systematic Reviews. Key websites and publications were handsearched. Studies conducted in Indigenous PHC which demonstrated some combination of CQI characteristics and assessed some aspect of implementation were included. A two stage analysis was undertaken. Stage 1 identified the breadth and focus of literature.

Stage 2 investigated barriers and enablers. The Framework for Performance Assessment in PHC (2008) was used to frame the analysis. Data were extracted on the study type, approach, timeframes, CQI strategies, barriers and enablers.

**Results:**

Sixty articles were included in Stage 1 and 21 in Stage 2. Barriers to implementing CQI processes relate primarily to professional and organisational processes and operate at multiple levels (individual, team, service, health system) whereas barriers to improved care relate more directly to knowledge of best practice and team processes that facilitate appropriate care. Few studies described implementation timeframes, number of CQI cycles or improvement strategies implemented and only two applied a change theory.

**Conclusion:**

Investigating barriers and enablers that modify implementation and impacts of CQI poses conceptual and methodological challenges. More complete description of CQI processes, implementation strategies, and barriers and enablers could enhance capacity for comparisons across settings and contribute to better understanding of key success factors.

## Background

Continuous quality improvement (CQI) programs have been taken up widely over the last decade by primary health care services caring for Aboriginal and Torres Strait Islander people in Australia [1] (henceforth referred to as Indigenous primary health care services). CQI programs use measurement and problem solving techniques to identify unwarranted variations in care and to test and embed improvements  [2, 3]. Key programs in Indigenous primary health care services have focused on improving outcomes in diabetes, cardiovascular disease, maternal and child health, rheumatic heart disease, health promotion, mental health and access to services [1].

Recent policy developments at the national level have shown a corresponding commitment to supporting CQI as part of routine primary health care delivery. Consultations carried out with Aboriginal health services as part of a national review of CQI confirmed widespread support for a national framework that could help services embed and sustain CQI processes in everyday practice. A 10-year, cross sector National CQI Framework for Aboriginal and Torres Strait Islander Primary Health Care 2015–2025 has been developed with investment from the Australian government of $40 million over three years to support uptake of the Framework within the Aboriginal Community Controlled (ACCHS) sector [4]. These developments place CQI firmly on the policy agenda.

Although there is a growing body of research about CQI both nationally and internationally, there has not yet been a systematic assessment of the achievements of CQI in Australian Indigenous primary health care services. International evidence shows that the effectiveness of CQI methods is variable [5], that implementation remains challenging, and that evidence about the extent to which contextual and other factors modify effects is limited [6]. We conducted a scoping review of the literature from studies of CQI in Australian Indigenous primary health care services to explore the breadth of literature and extent of uptake, barriers and enablers to implementation and impact. From this, we draw conclusions about the state of knowledge in Australia with a view to informing how future research and evaluation might be intensified to support implementation at the service level and enhance capacity for synthesising knowledge for policy and practice. The review is reported in two parts. This paper focuses on what has been learned about uptake, and about barriers and enablers to implementing CQI - the implementation study. A companion paper reports on impacts on service systems, care and client outcomes - the impact study [5, 7, 8].

## Methods

The review follows the scoping methodology outlined by Arksey and O’Malley [7]. It is the first step in a larger systematic review of the Australian and international literature on CQI programs in indigenous, ethnic minority and underserved populations (Gardner et al. in prep). Searches were conducted in MEDLINE, CINAHL and the Cochrane Database of Systematic Reviews to December 2016 using a combination of search terms relating to continuous quality improvement, primary health care, indigenous populations, ethnic minority populations and chronic disease (See Appendix). Additional hand searches of key Australian Indigenous research and CQI program websites (Lowitja Institute, Health Infonet, Menzies School of Health Research, the Kirby Institute, One21Seventy; Improvement Foundation; Queensland Aboriginal and Islander Health Council Close the Gap Collaborative; George Institute, Torpedo and Health Tracker), and snow balling of key authors was undertaken to locate additional articles, evaluation and other reports to December 2016, that were relevant to CQI in the Indigenous primary health care setting in Australia.

For both the implementation and impact studies, a nested, two-stage approach to analysis was undertaken. Stage 1 identified the breadth and focus of literature and Stage 2 explored barriers and enablers to implementation, impacts on service systems, care and outcomes. Following Sollecito and Johnson, [9] CQI was defined as “a structured organisational process for involving personnel in planning and executing a continuous flow of improvements to provide quality health care that meets or exceeds expectations” and includes a common set of characteristics of CQI identified in an international Delphi process [10]. To be included in the stage 1 analysis (common to both the implementation and impact studies), studies had to report on CQI programs or activities in Indigenous primary health care services that demonstrated some combination of these characteristics. Journal articles as well as evaluation and technical reports were included; fact sheets and policy briefs were excluded.

Separate stage 2 analyses were conducted for the implementation and impact studies. The Framework for Performance Assessment in Primary Health Care (FPA_PHC) [11] was used to frame our analysis. The framework distinguishes between measurement of improvements at the service level (Level 2), at the level of care received by patients (Level 3) and client outcomes (Level 4). For this implementation study, papers subjected to further analysis in stage 2 were those that investigated barriers and enablers to implementing CQI processes and to implementing changes in systems supporting improvements in care (Level 2 of the FPA_PHC). Studies and technical reports that did not report research directed to understanding barriers and enablers or reports that drew on data already reported in peer reviewed literature were excluded. This included studies in which the author/s reflected on the barriers and enablers underpinning observed changes and relationships without providing some data to support them. Where studies reported on barriers as part of assessing the quality of systems using a System Assessment Tool (SAT), only those that related specific barriers or enablers with SAT domains were included. Study protocols and publications in which the only approach to dealing with barriers and enablers was via review of literature were also excluded. Studies were also excluded if they did not specifically report on Indigenous services or clients.

Three researchers extracted data (KG, BS, MC). In Stage 1, studies were grouped into programs and classified according to the study type and focus, and whether they were evaluation or technical reports, or peer reviewed black literature. Black literature was further classified as either study protocols, history, feasibility or baseline studies; barriers and enablers; or impacts (service systems, care or client outcomes). In Stage 2, data for this implementation study were entered into a table that included details on the study design and approach; barriers and/or enablers to implementation of the CQI cycle; and barriers and/or enablers to implementing changes to service systems to improve care.

## Results

**Fig. 1:**
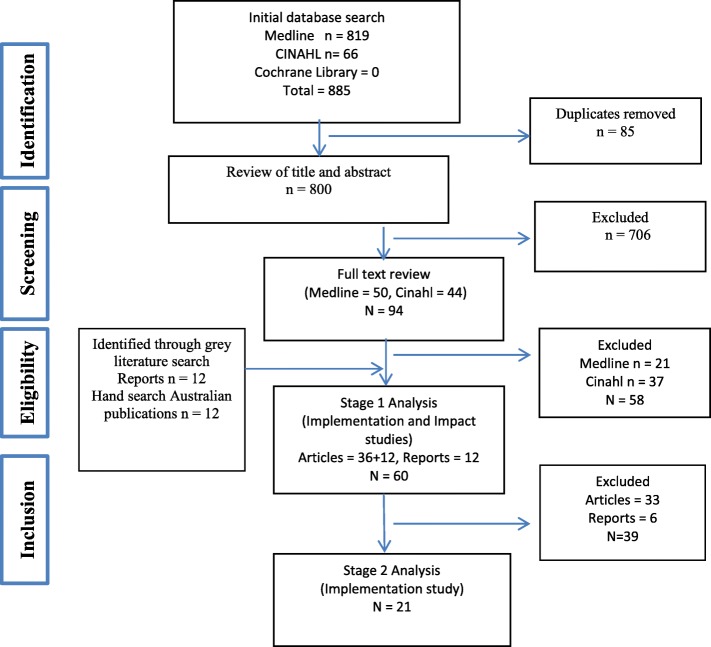
Search Process

The search results are summarised in Fig. 1. Eight hundred eighty-five articles were identified in the initial search of the black literature, and after exclusion of duplicates 800 were subjected to title and abstract review. A subset of 94 publications was then subject to full text review and assessed for eligibility for stage 1, resulting in 36 peer-reviewed publications. A further 12 reports (grey literature) and 12 publications were identified through the hand searching for inclusion in stage 1 (total = 60). Of these 21 were selected for stage 2 analysis for this implementation study (see below).

### Stage 1 analysis

The 60 publications included in stage 1 (both studies) (see Table 1) showed that the principal published program was Audit and Best Practice for Chronic Disease (ABCD) (2002 to 2005) and its extensions ABCDE (2005 to 2009), One21Seventy (2010–2016) and the ABCD Partnership (henceforth called the ABCD Group). Forty-two of the 60 publications [12–53] (70%) came from this group. Of the remaining 18, 1 is from the Australian Primary Care Collaborative [56], 5 are from the Aboriginal Community Controlled Health Services (ACCHS) sector [57–61], 8 are from research projects [54, 55, 62–66, 69] and the remaining 4 and are a review of the Northern Territory CQI investment strategy [67], a national appraisal [68], two reports associated with the national CQI Framework for Aboriginal and Torres Strait Islander Primary Health Care, namely recommendations for a national framework and the consultation draft of the Framework.

**Table 1:** Publications on CQI programs and activities in Indigenous primary health care services 2005 to 2016

	Program	CQI Focus/Topic	Grey literature N=12	Black literature N=48		
			Evaluation reports (E); Technical reports (T) n=12	History of CQI (H); Study protocols, descriptions, tools (P); Feasibility (F); Baseline (B); Did not report Indigenous Services (D); n=28	Barriers and enablers ^1^ n=15	Impact on service systems^2^ (S); care (C); client outcomes (O) n=14
	ABCD Group					
11.	Si, Bailie, Connors *et al.* 2005	Systems assessment for diabetes care		X (B)		
12.	Bailie, Si, O’Donoghue *et al.* 2007	Program description		X (P)		
13.	Bailie, Si, Dowden *et al.* 2007	Diabetes care			X	X (S,C,O)
14.	Bailie, Si, Dowden *et al.* 2007	Program report	X (E)			
15.	Si, Bailie, Dowden *et al.* 2007	Adult preventive services			X	X (S,C)
16.	Bailie, Si, Connors *et al.* 2008	Study protocol (Extension project)		X (P)		
17.	Bailie, Sibthorpe, Gardner *et al.* 2008	History		X (H)		
18.	Si, Bailie, Cunningham *et al*. 2008	Systems assessment for chronic disease care		X (B)		
19.	Bailie, Si, Dowden *et al.* 2009	Childhood immunisation				X (C)
20.	Bailie, Si, Shannon *et al.* 2010	Study protocol		X (P)		
21.	Gardner, Dowden, Togni *et al.* 2010	Program implementation			X	
22.	Rumbold, Bailie, Si *et al.* 2010	Maternal health		X (B)		
23.	Schierhout, Brands, Bailie 2010	Program report	X (E)			
24.	Si, Bailie, Dowden *et al.* 2010	Diabetes care		X (B)		
25.	Bailie, Si, Connors *et al.* 2011	Preventive		X (B)		
26.	Gardner, Bailie, Si *et al*. 2011	Program implementation			X	
27.	Rumbold, Bailie, Si *et al.* 2011	Maternal care		X (B)		
28.	Si, Dowden, Kennedy *et al.* 2011	Depression		X (B)		
29.	Gausia, Thompson, Nagel *et al*. 2013	Antenatal emotional wellbeing		X (B)		
30.	Ralph, Fittock, Schultz *et al*. 2013	Rheumatic heart disease			X	X (S,C)
31.	Schierhout, Nagel, Si *et al*. 2013	Depression in diabetes		X (B)		
32.	Schierhout, Hains, Si *et al.* 2013	Program implementation			X	
33.	Bailie, Matthews, Bailie *et al*. 2014	Care for children - report	X (E)			
34.	Matthews, Schierhout, McBroom *et al*. 2014	Diabetes care			X	X (C)
35.	O’Donoghue, Percival, Laycock *et al.* 2014	Health promotion		X (F)		
36.	Bailie, Matthews, Nagel *et al*. 2015	Mental health	X (E)			
37.	Bailie, Schierhout, Cunningham *et al.* 2015	Program implementation	X (T)			
38.	Bailie, Schultz, Matthews *et al*. 2015	Program implementation	X (E))			
39.	Gausia, Thompson, Nagel *et al*. (2015)	Antenatal mental health		X (B)		
40.	Gibson-Helm, Teede, Rumbold *et al.* (2015)	Antenatal care			X	X (S,C,O)
41.	Matthews, Connors, Laycock *et al*. 2015	Program report	X(E)			
42.	Newham, Schierhout, Bailie *et al*. 2015	Program implementation			X	
43.	Puszka, Nagel, Matthews *et al.* 2015	Youth health		X (P)		
44	Burnett, A., Morse, A., Naduvilath, T., Boudville, A., Taylor, H., Bailie, R. (2016)	Eye health		X (B)		
45	Schierhout, Matthews, Connors, *etal.* 2016	Diabetes				X ©
46	Cunningham, Ferguson-Hill, Matthews, Bailie. 2016	Systems Assessment Tool development		X(P)		
47	Gibson-Helm, Rumbold, Teede, Ranasinha, Bailie, Boyle 2016	Pregnancy care				X©
48	Bailie, Laycock, Matthews, Bailie 2016	Chronic illness			X	
49	Laycock, Bailie, Matthews, Bailie 2016	Evidence practice gaps		X(P)		
50	Percival, O'Donoghue,Lin, Tsey, Bailie 2016	Health promotion				X©
51	Bailie, Matthews, Bailie etal 2016	Preventive care		X(B)		
52	Vasant, Matthews, Burgess 2016	Cardiovascular		X(B)		
	Torres Strait Communities					
53.	McDermott, Schmidt, Sinha *et al*. 2001.	Diabetes care				X(S,C,O)
54.	McDermott, Tulip, Schmidt *et al.* 2003	Diabetes care				X (S,C,O)
	Australian Primary Care Collaborative (APCC)					
55.	Knight A, Caesar C, *et al.* 2012	Access, patient self-management, preventive care, diabetes, CHD, COPD		X (D)		
	QAIHC Closing the Gap Collaborative					
56.	QAIHC 2011		X (E)			
57.	Panaretto, Gardner , Button *et al.* 2012	Risk factor management, health assessments, hypertension, diabetes care			X	X (C,O)
	Kimberley Region ACCHSs					
58.	Marley J, Nelson C *et al.* (2012)	Diabetes care			X	X (C,O)
59.	Stoneman, Atkinson, Davey M et al 2014	Diabetes care		X (B)	X	
	Winnunga Nimmityjah					
60.	Dorrington, Herceg, Douglas *et al.* 2014	PAP smears			X	X (C)
	Torpedo/Health Tracker					
61.	Peiris et al 2012	Cardiovascular risk		X (P)		
62.	Patel, B, Patel A *et al.*	Cardiovascular risk		X (P)		
63.	Peiris 2015	Cardiovascular risk		X (D)		
	STRIVE					
64.	Ward J, McGregor S *et al.* 2013	Sexually transmitted infections		X (P)		
65.	Hengel B, Guy R, *et al.* 2015	Sexually transmitted infections			X	
	Miscellaneous					
66.	Allen and Clarke 2013	State evaluation report	X (E)			
67.	Wise M, Angus S *et al.* 2013	National appraisal	X (T)			
68.	Lowitja Institute 2014	National CQI Framework Recommendations	X (T)			
69.	Lowitja Institute 2015	National CQI Framework	X (T)			
70	Ralph, Read, Johnston, *et al* 2016	Rheumatic heart disease		X (P)		

^1^ Analysis reported in implementation study, Gardner *et al*.

^2^ Analysis reported in associated impact study, Sibthorpe *et al*.

The non-peer reviewed literature (*n* = 12) comprised 8 evaluations [15, 24, 34, 37, 39, 42, 57, 67] and 4 technical reports [38, 68]. In the black literature (*n* = 48), the majority of publications are descriptive and baseline studies (58%, *n* = 28) that include study protocols [13, 17, 21, 44, 47, 50, 62, 63, 65, 69], a history of CQI [18], a feasibility study [36] or baseline/single audit studies [12, 19, 23, 25, 26, 28–30, 32, 40, 45, 52, 53, 60] or studies that did not report specifically on Indigenous services or clients [56, 64]. One of the latter was a publication from the Australian Primary Care Collaborative [56], a major CQI program in Australian primary health care, that reported on changes for a completed 18-month collaborative over 13 ‘waves’ between 2005 to 2011 for 1132 general practices and 53 ACCHSs across Australia but results for the ACCHSs are not reported separately [56].

Fifteen black literature publications (31%) report on some aspect of barriers and enablers to implementation [14, 16, 22, 27, 31, 33, 35, 41, 43, 49, 58–61, 66]. Fourteen publications (29%) report on changes to service systems and/or care and and/or client outcomes - six (13%) on service systems [14, 16, 31, 41, 54, 55], all 14 on client care [14, 16, 20, 31, 35, 41, 46, 48, 51, 54, 55, 58, 59, 61] and six (13%) on client outcomes [14, 41, 54, 55, 58, 59]. Thus, among those studies reporting on client care, there have been as many baseline only studies as impact studies and there are as yet relatively few studies reporting on client outcomes. Only two publications from the ABCD Group reported changes in client outcomes to end 2016 [14, 41]. The other reports on outcomes came from the Torres Strait communities [54, 55], the QAIHC Collaborative [58] and Derby Aboriginal Medical Service [59]. Both Torpedo/Health Tracker [62–64] and STRIVE [65, 66] are in the early stages of their research and are yet to report on outcomes.

We know from experience and from the national consultation with Indigenous health services [1] that the published literature is a long way from capturing all the CQI activity taking place in this setting. With that important caveat in mind, this review shows that there has been very significant, though geographically uneven, uptake of CQI in Indigenous primary health care. The states/territories dominant in the literature are the Northern Territory and Queensland, with some activity in Western Australia and South Australia, in a small region in western NSW, and in the ACT. It is impossible to determine exactly how many services have participated in published studies but an earlier factsheet from the ABCD partnership (2015) indicated that 270 services had participated in One21Seventy between 2005 and 2014, of which 98 were ACCHS. This is a significant level of uptake among ACCHS but there are big gaps in knowledge about uptake in the private general practices and government clinics serving Indigenous clients and populations. To a large extent these findings reflect the reach of the ABCD program. Unfortunately, the paper from the Australian Primary Care Collaborative [56] does not provide any information that would shed light on general practices serving Indigenous populations, and nothing has been published about the APCC ‘Closing the Gap’ Collaborative so little is known about uptake for Indigenous primary health care in this sector (when Torpedo/Health Tracker and STRIVE report they will help to fill this gap).

### Stage 2 analysis – Implementation study

Six reports [24, 34, 37, 39, 42, 67] and 15 peer reviewed publications addressed some aspect of barriers and enablers [14, 16, 22, 27, 31, 33, 35, 41, 43, 49, 58–61, 66] and were included in Stage 2. Summary information on the key CQI strategies, study characteristics and barriers and enablers identified in these 21 publications are shown in Table 2. As shown, key strategies used in these CQI programs include annual audit cycles, use of key performance indicators, systems assessments, rapid PDSA cycles, information platforms for data analysis and reporting including comparisons with other services, and action planning.

**Table 2:** Barriers and enablers for published studies meeting eligibility criteria

	Authors	Study approach	Changes in Service Systems FPA_PHC Level 2	
			Barriers and enablers to implementing CQI	Barriers and enablers to implementing improvements in care (evidence - practice gap)
	ABCD Group A national service support program of annual PDSA cycles involving: • Manual clinical file audits (n=30 client records) for one or more of vascular and metabolic disease (diabetes, CHD, hypertension, renal disease); maternal health care; child health care, preventive services; mental health; rheumatic heart disease; health promotion; • Systems Assessment Tool (SAT), generic or specific to the file audit(s), covering the following domains: delivery system design; information systems and decision support; self-management support; links with the community, other health services and other services and resources; organisational influence and integration; • Web-based data entry and reporting system showing trends over time; comparison with audit data from other de-identified participating services; • Information feedback to service staff and an action planning workshop. Program training provided. Processes externally facilitated variably over time. Health service staff were responsible for implementing and documenting action plans. (*Key activities described in the black literature in Bailie, Si, O’Donoghue *et al. (*2007) and Bailie, Si, Dowden *et al*. 2007; and in the study protocol for the extension phase (Bailie, Si, Connors *et al.* 2008).			
13	Bailie, Si, Dowden *et al.* (2007) *Improving organisational systems for diabetes care in Australian* *Indigenous communities*	Study period 2002-2005; NT (Top End); purposive sample of 12/53 services in the Top End (mix of community controlled, government, health board); baseline plus 2 annual follow-up cycles. Diabetic clients (total =295) with annual follow up of the same clients. All services completed all cycles. Comprehensive list reported of examples of improvement strategies implemented across the 12 services categorised according to SAT domains; strategies not linked to services/changes in SAT scores.		At 2 years, statistically significant improvement in median scores for all 7 SAT domains. Reflections on barriers and enablers to improve care: Barriers to improved care appeared to be related to inadequate attention to abnormal clinical findings and medication management. Enablers: Improvement in intermediate outcomes may be achieved by addressing system barriers to therapy intensification through engagement of medical staff in CQI activities and/or greater use of nurse-practitioners.
15	Si, Bailie, Dowden et al.. (2007) Delivery of preventive health services to Indigenous adults: response to a systems-oriented primary care quality improvement intervention	Study period 2002-2005; NT (Top End); purposive sample of 12/53 services in the Top End (mix of community controlled, government, health board); baseline plus 2 annual follow-up cycles. Process as for Pub #1 but clinical audits were for random samples (n=30) of clients with no known diagnosis of chronic disease (total = 360) and follow-up audits were new samples. All services completed all cycles. Some examples of improvements strategies across the 12 services were classified with respect to SAT domains but not linked to services/changes in SAT scores. At 2 years, “Marked improvements across each [SAT] system component over the study period”; statistical significance not reported. Statistically significant improvements in counselling services were achieved over 2 audit cycles but no change in preventive care such as measurement of waist circumference, blood pressure etc.		Barriers to improvements in preventive care appeared to be related to a limited focus on improving service systems most likely to influence change eg. “external linkages” (outreach and health promotion type initiatives) and “organisational influence” (use of management processes to demonstrate interest in preventive care and securing new resources) Enablers for achieving improved counselling in diabetes care were a focus on systems likely to influence change eg. delivery system design (use of interpreters and revision of team roles); decision support (training by visiting specialists).
21	Gardner, Dowden, Togni (2010)	Study period: First year of participation in ABCDE for 61 services (35 ACCHSs; 26 Govt) in NT, WA, NSW, QLD over the period 2006-2008. Data included routinely collected regional and service profile data; uptake of tools and progress through the first CQI cycle, interviews with key stakeholders (n=48). Organising framework for data analysis was the Greenhalgh diffusion of complex innovation framework which identifies attributes of the intervention and the change agency; process of diffusion; elements of user system and the outer system context.	Enablers: supportive policy environment for CQI; compatibility of CQI tools with MBS incentives; individual motivation for improvement processes; leadership support endorses & provides authority to take up CQI; skills; organisational networks; high level committee oversight within organisations; coordinator position responsible for implementation; clinical staff involvement; information infrastructure; networking, training and facilitation of CQI provided by ABCD team; Indigenous, academic and clinical champions promote understanding of how CQI contributes to organisational, professional and community objectives; Barriers: high staff turnover & shortage impeded implementation of CQI cycle; lack of leadership; lack of oversight for implementation; few organisational networks; sudden changes in staffing, leadership; community priorities	Not discussed
23	Schierhout, Brands, Bailie (2010)	ABCDE Project Final report 2005-2009 investigates acceptability of the ABCD model in 12 Aboriginal Primary Health Services in the NT. Report draws on the data derived from purposively structured dialogue with hub co-ordinators to explore perceptions of the degree to which key influences on engagement were operating within each health centre in each year of participation; and analysis of more than 48 supplementary in-depth interviews with practitioners, health centre managers and staff, policy makers, hub co-ordinators and researchers conducted as part of a PhD project aligned with the ABCD Extension project (Gardner et al. 2010). No theory reported in this report but reported in Gardner 2010.	Enablers at: Service level include commitment by senior management; planned implementation that linked CQI to organisational aims and adaptation to local needs; improving record keeping of clinical data; allocating time and resources for staff to participate in CQI; investing in professional development in CQI. Regional level: High level commitment from health authorities and organisation wide networks	Enablers: Larger and better resourced health services, those under a regional health authority and those with engaged clinical leaders were more likely to achieve improvements. Enablers include regional level management support; adequate levels and stable staffing; involvement of AHWs in clinical care and CQI; completion of CQI processes according to project protocols.
26	Gardner, Bailie, Si etal (2011)	Review paper drawing on ABCD papers and other published evidence.	Barriers: staff turnover, poorly aligned data capture systems, lack of appropriate services for referral Enablers: a clear internal vision and purpose for which the ABCD quality tools and processes would be used and which adopted a strong regional approach to supporting services in data analysis and response to problems that lie beyond the capacity of individual services to solve	
30	Ralph, Fittock, Schultz *et al*. (2013)	Study period 2008-2010; NT (Top End and Central Australia); 6 services (sampling strategy not reported; jurisdiction not reported); baseline plus 2 annual follow-up cycles. Process as for Pub #1 but audits for all clients with RHD at each cycle (n=154, 145,156) (new samples).All services completed all cycles. Participatory action methods included facilitated discussion with primary care staff aided by Systems Assessment to identify system barriers to high quality care. Improvement strategies such as improved record-keeping, triage systems and strategies for patient follow-up encouraged but strategies for 6 participating centres not reported.		SAT domain organisational influence and integration improved over 3 years, and appeared to be related to performance in BPG prophylaxis. However tests of significance were “not calculated given the somewhat subjective nature of these scores ….” Variation in contextual characteristics of 6 health centres included population size; geography; accessibility; staffing; record keeping; and governance arrangements; mobile populations; number of RHD deaths; ability to locate files. Wide variation in key performance measures including recording eg.% clients receiving routine injections and % people with documented risk classification.These not discussed specifically in relation to implementation
32	Schierhout, Haines etal (2013)	Study period 2002-2012; data obtained from 36 health centers completing 3 or more annual cycles, quarterly project reports, and workshops with 12 key informants who had key roles in project implementation. Aim was to abstract context-mechanism-outcome configurations and from those develop strategies to strengthen the program.		Three mechanisms were identified: collective valuing of clinical data for improvement purposes; collective efficacy; and organizational change towards a population health orientation underpinned “successful CQI” as measured by improvements in the delivery of diabetes and preventive care. Strong central management of CQI and alignment of CQI with local priorities were favourable contexts for collective valuing of clinical data. Positive experiences of collaboration led to collective efficacy. Strong community linkages, staff ability to identify with patients, and staff having the skills and support to take broad ranging action, were favourable contexts for the mechanism of increased population health orientation
33	Bailie, Matthews, Bailie (2014)	Study period for audit data 2007-2013; 10,000 clinical audits in 132 centres; NT, QLD, SA,WA, NSW. A 3 phase consensus process was used to identify priority evidence-practice gaps in child health care, based on these data. The purpose was to stimulate discussion and enhance ownership of the development of interventions to address system gaps. Key gaps identified included recording of immunisations; monitoring & recording key measures and abnormal findings; recording advice & brief interventions; recording enquiries on tobacco & alcohol use; systems to support links with communities & regional centres		Barriers and enablers to high quality care include Staffing/workforce support recruitment & retention; staff shortage; development of clinical information systems; community engagement and health literacy; training and development to support skills for provision of best practice care.
34	Matthews, Schierhout, McBroom *et al.* (2014)	Study period 2005-20012; NT, Q, NSW, SA, WA; 132 services participating in One21Seventy/ABCD Program (73% government, remainder community controlled). Clinical audits over 7 years of random samples of clients with diabetes (n=10,674 client records); cycle completion rates: baseline only (32 services) 1-2 cycles (55 services), ≥3 cycles (45 services); audits conducted by services with training and support provided; SAT, feedback workshops and action planning and improvement strategies implemented not discussed. Process indicators of quality of care for each patient were calculated by determining the proportion of recommended guideline scheduled services that were documented as delivered. Multilevel regression models used to quantify amount of variation in Type 2 diabetes service delivery attributable to health centre or patient level factors and to identify those factors associated with greater adherence to best practice guidelines.		Health centre factors explained 37% of the differences in level of service delivery between jurisdictions with patient factors explaining only a further 1 Health centre factors that were independently associated with adherence to best practice guidelines included: • longer participation in the CQI program, • remoteness of health centres, • regularity of client attendance. Significantly associated patient level variables included • greater age, and • number of co-morbidities • disease complications.
36	Bailie, Matthews, Nagel (2015)	Study period for audit data 2009-2014; 975 clinical audits & 29 SATs in 21centres; NT, QLD, SA,WA,NSW. A 2 phase consensus process involving 13 stakeholders was used to identify priority evidence-practice gaps in mental health care, based on these data. The purpose was to stimulate discussion and enhance ownership of the development of interventions to address system gaps.		Key evidence practice gaps identified: consistent recording of client health summaries; enquiry & recording of risk factors & brief interventions; consistent recording of scheduled services; follow up of abnormal results; health centre systems, particularly links with the community to inform service and regional planning; organisational commitment for structures and processes that promote safe, high quality care, and team structure and function.
38	Bailie, Schultz, Matthews (2015)	Priority evidence-practice gaps and stakeholder views on barriers and strategies for improvement preventive health care		
40	Gibson-Helm, Teede, Rumbold et al. (2015)	Study period 2007-20012; NT, QLD, NSW, SA, WA; 76 services participating in One21Seventy/ABCD Program Research Partnership (65% government, remainder community controlled). Clinical audits of clients who had recent pregnancy in up to 4 cycles; audits conducted by trained internal or external personnel with regional support; Systems assessment tool (SAT) externally facilitated; feedback workshops and action planning noted but not discussed. Improvement strategies not linked to SAT.		In 21 services statistically significant associations found between 3/6 SAT scores and diabetes screening; 1/6 SAT scores and B/P first trimester. 0/6 SAT scores and BMI and B/P at any time Health centre system enablers: more highly developed PHC information systems and decision support enable first trimester BP screening; more highly developed PHC systems for self management support and organisational influence and integration
41	Matthews V, Connors C etal 2015	Study period 2005-13;18,000 clinical records; 160 PHC centres A three phased process engaged 380 stakeholders from Aboriginal and Torres Strait Islander PHC centres and systems in analysing and interpreting, chronic disease audit data. A consensus process was used to identify priority evidence-practice gaps in chronic illness care, barriers and enablers to high quality care; system-wide strategies for achieving improvement based on these data. The purpose was to stimulate discussion and enhance ownership of the development of interventions to address system gaps.		Enablers for improving practice evidence gaps in CD include: follow-up of abnormal findings; adherence to treatment guidelines; assessment and support of emotional well-being for patients with CD; improved vaccination coverage; links between services; workforce recruitment, retention, capacity and training; capacity to provide patient centred care; modification of AHW roles; community involvement and participation in service delivery design; develop CQI culture, health literacy and leadership. Barriers to high quality care include workforce recruitment and retention; capacity to provide patient-centred care; community engagement and participation in service delivery design; training and development of health centre staff and management.
42	Newham J, Schierhout Getal 2015	18 semi-structured interviews in 11 Aboriginal primary health-care services in South Australia	Barriers at the macro level include resource constraints and access to project support; meso level include senior level management and leadership for quality improvement and the level of organisational readiness; at micro level include knowledge and attitudes of staff, resistance to change and lack of team tenure. Enablers include training, someone who drives the CQI process at the service, organisational and individual change, a regional approach,	
48	Bailie, Laycock, Matthews, Bailie 2016	Evidence practice gaps identified using audit data 2012-13 for chronic illness care ( 123 health centres; 6523 patient records and 90 SATs) and for child health care ( 94 health centres; 4011 patient records, 62 SATs) together with data derived from purposively structured dialogue with stakeholders and a survey to rank the relative importance of areas of poor recording, delivery of care and health centre systems		Seven priority evidence-practice gaps were identified for chronic illness care and five for child health Common gaps were related to follow-up of abnormal findings; recording of advice on risks to health; and systems for links between health centers and communities. Respondents felt that health center and system attributes were of greater or equal importance compared to staff attributes in improving quality of care. 5 primary drivers and 11 secondary drivers of high-quality care are identified.
	QAIHC Closing the Gap Collaborative An ACCHS state affiliate member service support program involving monthly automated extraction from electronic health records of aggregated data for 21 indicators of overall service performance (‘QAIHC core indicators’) with analysis and web-based reporting to participating services. (Additionally, described in the grey literature only, were three quality improvement support coordinators, a network of quality improvement support officers, 2-day learning workshops every 6 months, face-to-face and web-based training seminars, an electronic discussion forum and a monthly electronic newsletter (QAIHC 2011).			
57	Panaretto, Gardner, Button et al. (2013)	Study period June 2010 - February 2012; QLD; 22 member services of Queensland Aboriginal and Islander Health Council (100% community controlled). Data available for a total of 19,727 regular clients, aggregated data reported for 5 time points. CQI processes, including state-wide ‘collaboratives’ not described. Improvement strategies implemented by health services not reported.	Not discussed	Contextual factors at the service level that may drive variation in improvement on performance: Clinical activities versus EPC items: One person activity versus team activity Interservice variation: SEIFA, community size and percentage of indigenous people in catchment. Remoteness ICAC or SAT scores: available staffing/workforce. Senior medical officer turnover. Ratio of doctors to patients workload per clinician Use of data platforms–Pen CAT usage or similar. APCC portal usage. Use of Plan Do Study Act cycles: CQI programme/collaborative Incentives: Staff flat salaries or incentives Patients: Staff and patients
	Derby Aboriginal Health Service A study in one health service of diabetes care and outcomes involving a retrospective audit covering a 10 year period during which time the service participated in CQI activities through ABCDE and APCC (time periods for involvement unclear).			
58	Marley J, Nelson C, O’Donnell V et al. (2012)	Study period 1999-09; WA; 1 service (community controlled). Retrospective audit of records of clients with diabetes (n=254 clients). CQI processes not described; Improvement strategies implemented by health services not reported. Consideration given to enablers for CQI through participant observation.	Service level enablers: Stable governance, community elected board, electronic health info system, consistency of senior staff, long term employment of Aboriginal Health Workers and Nurses; CQI approaches based on a culture of organisational appraisal and improvement; encouraging review and reflection among staff at all levels; embracing change in response to gaps; CQI and formalisation of regular internal and external audit; regional support & standardisation of processes	Enabling policies identified: reimbursement for health checks and for chronic disease management plans and follow up; access to low/no cost medications in remote areas
	Kimberley Services, 2011-2012 A study in 4 ACCHS in Western Australia of diabetes care involving a retrospective audit of records for Aboriginal and Torres Strait Islander primary care patients aged ≥15 years with a confirmed diagnosis of T2DM at four Kimberley ACCHSs from 1 July 2011 to 30 June 2012. Interviews with health service staff and focus group discussions with patients post audit.			
59	Stoneman (2014)	Study period 1 July 2011 to 30 June 2012; Kimberley WA; 4 Services (community controlled). Retrospective audit of records for patients aged ≥15 years with a confirmed diagnosis of T2DM (n=348 patients). Interviews with 19 staff (9 AHWs, 7 RNs, 3 GPs) from 4 ACCHSs after seeing audit results. 3 focus groups with 16 patients from 3 ACCHSs. Thematic analysis	Seamless and timely data collection; local ownership of CQI process; openness to admitting deficiencies and willingness to embrace change; regional CQI facilitator.	Enablers included: clearly defined staff roles for diabetes management; increased role for AHWs in chronic disease management including training in self management approaches, retinal camera & point of care HbA1c; efficient recall systems & involvement of AHW or Aboriginal outreach worker in recall; well-coordinated allied health services; increased staffing to increase focus on chronic disease; guidelines and staff training to use Mmex; whole service involvement interpreting audit results; staff and community involvement in developing improvement strategies. Barriers include high staff turnover, lack of clarity over responsibility for recall; uncertainty of how to use Mmex for recall.
	Winnunga Nimmityjah Aboriginal Health Service A study in one health service of Pap smear screening involving a baseline retrospective clinical audit, survey of convenience sample of clients (n=32), focus groups with staff and client Women’s Group, rapid PDSA cycles and follow up clinical audits.			
60	Dorrington, Herceg, Douglas *et al.* (2015) *Increasing Pap smear rates at an urban Aboriginal Community Controlled Health Service through translational research and continuous quality improvement*	Study period 2009-2013; ACT; 1 service (community control). Baseline audits for eligible women (n=213), 5 rapid PDSA cycles (4-5 wks duration) in 2012, survey of convenience sample of clients (n=32), follow-up assessment of annual screening rate compared with years 2009-2011. Comprehensive description of CQI processes: 1) Baseline data collection tool implemented as first PDSA 2) Promotional material used to raise client awareness of Pap smear screening. 3) Afternoon clinic for health appointments with a female GP established. 4) Pap smear recall system reviewed and cleaned. 5) Reminder letter updated to include specific information about cervical cancer in Aboriginal and Torres Strait Islander women; mail-outs included a culturally appropriate leaflet. 6) Education provided to the Social Health Team to facilitate discussions with clients about Pap smear screening	nil	Barriers to screening identified by clients included forgetting, not having time and being too busy; discomfort; not liking smears; fear of results; shyness and embarrassment; not knowing which professional to see; other health issues; chronic conditions consuming consultation time. Enablers were GP prompts, appointments, reminders (letters; text messages)
STRIVE				
65	Hengel, Guy etal 2015	Study period: 36 in-depth interviews in 22 out of 65 health centres across four regions in northern and central Australia participating in a randomised control project on STIs.		Barriers including Aboriginal cultural norms that require the separation of genders and traditional kinship systems that prevent some staff and patients from interacting. Both were exacerbated by a lack of male staff. Other common barriers were concerns about client confidentiality (lack of private consulting space and living in small communities), staff capacity to offer testing impacted by the competing demands for staff time, and high staff turnover resulting in poor understanding of clinic systems. Strategies, such as team work, testing outside the clinic and using adult health checks were used to address these barriers.
66	Allen and Clarke 2013 *Evaluation of the NT CQI Investment Strategy*	Study period 2009-2013. NT. External evaluation drawing on review of evidence, key informant interviews; five case studies; review of program data and key documents; sense making workshop.	Key barriers relate to geographical remoteness; cultural diversity and the influence of social determinants on health outcomes. Other challenges include a high turnover of the health workforce, and significant expansion and reform of the health system.	

Of the included studies, 7 reported barriers and enablers to implementing CQI processes [22, 24, 27, 43, 59, 60, 67] and 17 studies reported on barriers and enablers to implementing changes to service systems to improve care [14, 16, 24, 31, 33–35, 37, 39, 41, 42, 49, 58–61, 66]. Three studies [24, 59, 60] reported both. Overall, the majority of papers (*n* = 14) are from the ABCD group with the remaining 7 papers coming from initiatives in the Indigenous sector [57–61], a research project [66] and an evaluation of the Northern Territory CQI [67].

#### Barriers and enablers to implementing CQI processes

Of the 7 studies that assessed barriers and enablers to implementing CQI processes, five [22, 24, 27, 43, 60] used in-depth interviews with stakeholders as a primary data source; one drew on author observations of measured changes in audit results [59]; and one was a multi-method evaluation drawing on interviews, focus groups, case studies and program data and documents [67]. Only one [22] used an explicit theory of change. Four studies were from the ABCD group, one each from the Kimberley region and Derby community controlled health services CQI programs and one an evaluation of the Northern Territory CQI investment strategy.

Across all studies, barriers and enablers were found at multiple levels of organisation: individual staff, team, organisation, region and the broader policy context. Implementing PDSA cycles into routine practice and integrating these into organisational and professional systems was found to be challenging despite widespread support and enthusiasm for CQI across the service sector. Commonly reported barriers included knowledge and attitudes of staff, resistance to change, difficulties in engaging some professional groups (general practitioners and middle managers), lack of team tenure, high staff turnover and insufficient senior management support and poor IT capacity [22, 27, 43, 67]. Teams often experienced difficulties in quarantining time for CQI and required assistance with data entry, information systems and technical expertise for data analysis and synthesis [22]. Manual audits were time-consuming and high levels of staff turnover in some services slowed implementation. Engagement of health service managers was critical to ensure that action plans were implemented into changes in service delivery. Where managers perceived the scope for making changes to organisational policies and procedures was limited or difficult, system redesign and actions for improvement were less likely to occur [22]. At the state wide level, the Northern Territory evaluation identified additional barriers related to geographical remoteness; cultural diversity; the influence of social determinants on health outcomes; and significant expansion and reform of the health system.

Conversely, commonly reported enablers included regional support and CQI facilitation and strong leadership. Schierhout’s report on the ABCDE project [24] identified service level enablers as commitment by senior management; planned implementation that linked CQI to organisational aims and adaptation to local needs; improved record keeping of clinical data; allocation of time and resources for staff to participate in CQI and investment in professional development in CQI. Stoneman [60] found that seamless and timely data collection; local ownership of CQI process; openness to admitting deficiencies; and willingness to embrace change were key enablers. Stable governance, community elected board, organisational commitment, strong leadership from senior and executive staff, clear delineation of staff responsibilities and objectives for CQI were also found to be critical [59]. Gardner [22, 27] and Newham [43] found that adequate provision of training and support, a no-blame systems oriented approach, well-established information and administrative systems, staff expertise in conducting audits and/or interpreting audit data, and an incremental approach to incorporating CQI activities into service routines were key enablers. Where clinic managers used CQI to underpin business planning processes, this helped to embed CQI processes [22]. At the regional level high level commitment from health authorities and organisation wide networks enabled CQI and at the policy level, Gardner et al. [22] found alignment of data collection and performance reporting processes reduced the burden on services of multiple collections and reporting arrangements.

#### Barriers and enablers to improving care processes

A variety of methods were used to assess barriers and enablers to improving systems supporting direct care delivery. Five ABCD reports [24, 34, 37, 39, 42] and two published papers [33, 49] collected qualitative data from purposively structured dialogues with stakeholders on their perceptions of the “evidence-to- practice gaps” underlying patterns of care reported in audits. Reports focused on chronic illness and preventive care [24], child health [34], mental health [37], preventive care [39] and chronic illness [42]. Reported barriers were similar across health topics and included staff shortages, poor follow-up of abnormal results, under-developed clinical information systems, lack of community engagement, poor health literacy, and inadequate training to support best practice care.

A further five ABCD studies identified barriers and enablers to care through the use of a systems assessment tool (SAT) [14, 16, 31, 35, 41]. The SAT is a measurement tool that assists staff to assess the level of development of their primary health care service systems across five domains: delivery system design, self-management support, decision support and clinical information systems, external linkages, and organisational influence and integration.. It is administered through a facilitated staff dialogue delivered as part of Step 3 of the annual CQI cycle. A consensus score is decided for each item in each domain using a score ranging from 0 to 11. The scores are subdivided into four categories defined as ‘limited or no support’ (0–2), ‘basic support’ (3–5), ‘good support’ (6–8) and ‘fully developed support’ (9–11). Brief descriptors help staff decide the score that best reflects their service systems.

Barriers to implementation identified in studies using the SAT were as follows. A 2007 study of diabetes care [14] found that inadequate attention to abnormal clinical findings and medication management were key barriers to improvements in care, leading the authors to recommend intensification of therapy through engagement of medical staff in CQI and greater involvement of nurse practitioners. A study in the same year on the delivery of preventive care [15] found that barriers were mainly related to service external linkages including outreach and health promotion activities, and others such as securing resources related to organisational influence. Enablers were in the delivery system design domain and included use of interpreters and revision to team roles, as well as training by visiting specialists (decision support). In Ralph’s study of rheumatic heart disease [31] barriers to improving care related to performance in administering prophylactic medication. Gibson Helm et al. [41] identified enabling factors for metabolic screening during pregnancy as including good information systems and good decision support systems which enabled first trimester BP screening and self-management support.

A mixed method realist review that sought to identify key mechanisms for change in achieving improvements in chronic disease and preventive care in the ABCD group [33] found that services in which there was collective valuing of clinical data for improvement purposes, collective efficacy and organisational change towards a population health orientation were more inclined to experience improvement. Health centres with strong central management of CQI, and those in which CQI efforts were locally driven and adapted to suit local priorities supported collective valuing of clinical data. Key mechanisms were collective efficacy and increased population health orientation. Strong community linkages, identification with patients, and staff skills for broad ranging action, were favourable contexts for population health orientation.

Through a quantitative analysis of change over time in key indicators, Panaretto et al. [58] identified factors that may drive variations in performance in community controlled services participating in the Queensland Aboriginal and Islander Health Council program. While these are referred to as “contextual factors” (consistent with quantitative methodology) rather than “barriers and enablers” (consistent with qualitative methodology), the factors overlap with those identified in other studies. They included the nature of the clinical activity (individual verses team arrangement), characteristics of the community such as size, Socio-Economic Indexes for Areas (SEIFA), remoteness and percentage of Indigenous people in the catchment; patient characteristics; quality of service systems or staffing/workforce issues such as ratio of doctors to patients; use of data platforms, PDSA program type, staff salary or incentives used.

Stoneman et al. [60] and Dorrington et al. [61] both conducted interviews with staff and clients to assess barriers and enablers to diabetes care and pap smears respectively. Stoneman found that optimal diabetes care was facilitated by clearly defined staff roles for diabetes management, support and involvement of Aboriginal Health Workers, efficient recall systems, and well-coordinated allied health services. Effective CQI features included seamless and timely data collection, local ownership of the process, openness to admitting deficiencies and willingness to embrace change. Dorrington identified patient barriers such as forgetting, lack of time, fear, shyness and the time taken by chronic disease. Enablers were GP prompts, reminders and appointments. Marley [59] identified enabling policies in a reflection on audit results finding that reimbursement for health checks and for chronic disease management plans and follow up; access to low/no cost medications in remote areas were primary enablers of improved care.

Hengel, Guy et al. [66] identified barriers to offering and conducting STI testing using interviews with 36 staff in 22 health centres in WA. These included Aboriginal cultural norms that require the separation of genders and traditional kinship systems that prevent some staff and patients from interacting. Both were exacerbated by a lack of male staff. Other common barriers were concerns about client confidentiality (lack of private consulting space and living in small communities), staff capacity to offer testing impacted by the competing demands for staff time, and high staff turnover resulting in poor understanding of clinic systems. Strategies, such as team work, testing outside the clinic and using adult health checks were implemented to address these barriers.

## Discussion

Studies of the barriers and enablers to implementation of CQI cycles and to the systems supporting improvements in care delivery have relied primarily on qualitative data collections, used either as a sole method or as part of mixed method designs drawing on analyses of audit data or measurement of improvements in service systems (SAT). Results from these studies indicate that barriers to implementing CQI relate primarily to professional and organisational change processes and operate at multiple levels (individual, team, service, health system), whereas barriers to improved care relate more directly to knowledge of best practice and team processes that facilitate appropriate care such as multidisciplinary teamwork for complex conditions, adequate staffing, follow up of care and linkages with communities, indicating a population approach, as well as financial incentives that support best practice.

While there is some overlap and possibly some conflation within some studies of these different factors, reported barriers and enablers are largely consistent across studies. The key barriers to implementing CQI in the studies reported here - time, staff turnover, training, teamwork, technical skills and organisational support - are also consistent with those reported internationally in CQI programs serving Indigenous and minority populations [70–75].

While some of the studies reviewed provided significant detail of implementation timeframes, number of PDSA cycles undertaken, improvement strategies implemented and support provided for implementation, none recorded details of the aims of the PDSA cycle itself, adaptations made to improvement strategies under the “do” and “study” parts of the cycle, what impacts were observed or what was embedded in the final “act” part of the cycle. CQI is based on small steps of change theory [2] and capturing data that reflects the iterative nature of change is important for developing a comprehensive picture of strategies that were trialled and found by services to be effective and those that were not.

In addition, few qualitative studies employed explicit theoretical approaches to inform the collection or analysis of data. It is well understood that CQI programs are complex interventions with multiple interconnected parts that are not only often difficult to define and describe [4], their implementation is challenging and impacts in health settings are highly variable [5]. To decide whether or not to carry out a CQI process, practitioners need to understand whether what works in one setting might work in another and thus research needs to examine the conditions for success [76]. Two studies in the ABCD group employed theories of change to explore the contextual and implementation arrangements that impeded or enhanced uptake and influenced service improvements [22, 33], thus moving some way towards adopting research strategies that could identify conditions for success.

As the spread of CQI programs across different organisational settings and community contexts continues under the proposed National CQI framework, it will be important to extend the current focus of research to incorporate the use of theory and methods capable of exploring whether findings from research in one setting can apply to another and therefore to inform the practice of CQI as it becomes routine activity in primary health care. There are three key challenges related to this endeavour - documenting the implementation of CQI activities themselves (e.g. steps taken in PDSA-type cycles); documenting the strategies tested and embedded as a consequence of those activities; and documenting elements of context. The first and third of these challenge are taken up here, the second is dealt with in our companion paper [8].

Firstly, adopting an accepted definition of CQI such as the one developed by Rubenstein and colleagues [10] could help to standardise documentation of CQI strategies and provide guidance to services on what information to collect. According to this definition, CQI involves systematic data guided activities, iterative development and testing process (Plan-Do-Study-Act cycles); designing with local conditions in mind; aiming to change routine work processes; multidisciplinary teams; specific predefined aims; sets of specific changes; using evidence relevant to the problem and data feedback to implementers. At a minimum, data on team composition, aim of the CQI endeavour, data sources and feedback processes, the specific change strategies and adaptations made over time and their observed impacts would provide the depth of information needed to support comparison of processes across services.

Identifying and describing relevant contextual factors is also essential for helping practitioners to determine whether or not to trial a specific CQI process in their service. Identifying context can be difficult and somewhat subjective [6, 76]. Described as “all factors that are not part of a quality improvement intervention itself,” [77], barriers and enablers are themselves often contextual factors, sometimes part of the implementing organisation (eg, information technology, team processes, leadership) sometimes external to it (eg, financial incentives, regional support structures) and sometimes part of the intervention itself. Although distinguishing between factors related to the CQI process itself and to the context in which it occurs may sometimes be blurred, improving analysis and recording of contextual factors will be an essential part of building a profile of comparative studies that help to establish which strategies are effective in which circumstances. Many frameworks are available to guide researchers [77–79]. Lau et al.’s 2016 four-level framework [77] distinguishes external contextual factors (policies, incentivisation structures, dominant paradigms, stakeholders’ buy-in, infrastructure and advances in technology) from organisation-related factors (culture, resources, integration with existing processes, relationships, skill mix, teamwork and staff involvement) from individual level factors (professionals, professional role, underlying philosophy of care and competencies) and from the characteristics of the intervention that impact on implementation (evidence of benefit, ease of use, adaptability to local circumstances). The application of mid-range theories to investigate the reasoning and resources required to operationalise CQI will help to provide further understanding of key mechanisms for change across different settings.

This study also found that contextual factors (otherwise called barriers and enablers) related to the implementation of CQI are distinct from those related to service systems supporting improved care. Making this distinction helps services struggling with different aspects of organisational change to identify where actions are required and the strategies that might best be used to achieve improvements. Our experience of working with different organisations indicates that some services that have implemented CQI with ease have struggled to achieve improvements in care.

In addition, the studies reviewed here show there is uncertainty about the utility of the SAT as a measurement tool but consensus on its benefits as a service development process for supporting team dialogue needed for action planning and implementation [31, 47]. It may be useful for future studies to draw on validated instruments to measure changes in contextual factors operating within implementing organisations that are important for CQI - teamwork, leadership and systems thinking [80] and use the SAT, which captures the functional aspects of service management, as a tool to support dialogue within teams implementing change strategies.

Finally, further work is required to embed qualitative approaches within quantitative designs that incorporate comparison groups to enhance the strength of evidence. Without solid evidence of the effectiveness of CQI, informing CQI policy, investment, national, regional and local program development will remain uncertain.

## Conclusion

Investigating the barriers and enablers which modify the implementation and impacts of CQI programs poses conceptual and methodological challenges. This review found a high level of consistency in reporting across studies but also identified differences in the barriers and enablers related to implementing CQI and those related to achieving change in service systems for improving care. Two main areas in which qualitative research could be expanded to achieve more complete documentation of factors that shape the success of CQI programs are discussed. Until research more fully describes the elements of CQI programs, their implementation and context, it will be difficult to compare findings across settings to identify key success factors that could inform broader roll-out of CQI programs. To achieve this, there is a need to move beyond the current descriptive focus of the qualitative research reviewed here to adopt more theoretically informed approaches. A number of theories and approaches are discussed. Embedding these in quantitative research designs which include comparison groups should enhance understanding of program components and mechanisms, the scope and depth of implementation as well as the impact of programs on service delivery and client outcomes which is needed to help inform consideration of where and how evaluation and research should be directed to best support program development and sustainability into the future.

## Appendix

**Table 3:** Search terms

1.	exp Quality Improvement/ or exp. Quality Assurance, Health Care/ or exp. Quality Indicators, Health Care/ or exp. “Quality of Health Care”/
2.	(quality improvement$ or improv$ quality or quality management$ or improv$ patient care).af.
3.	1 or 2
4.	exp efficiency, organizational/ or exp. organizational innovation/ or exp. models, organizational/ or exp. organizational objectives/ or exp. decision making, organizational/ or exp. Total Quality Management/
5.	(organi$ intervention$ or organi$ efficiency or organi$ chang$ or organi$ innovation$ or organi$ structur$ or organi$ model$ or organi$ system$ or organi$ strateg$ or organi$ cultur$).af.
6.	(rapid cycle or PDSA or plan do study act or PDCA or plan do check act or plan do check adjust or lean management or six sigma or audit feedback or total quality management or tqm or clinical governance or chronic care model or mbqa or malcolm baldrige quality award or efqm or european foundation quality management or accreditation or decision support or medical audit or clinical audit or guideline adherence or benchmark$).af.
7.	(staff attitude$ or staff management or staff relation$ or staff training or staff education or staff development or personnel attitude$ or personnel management or interprofessional relation$ or personnel training or personnel development or cultural awareness or cultural safety or opinion leader$ or champion$ or teamwork$).af.
8.	4 or 5 or 6 or 7
9.	exp Health Services, Indigenous/ or exp. United States Indian Health Service/ or exp. Primary Health Care/ or exp. Family Practice/ or exp. General Practice/ or exp. Physicians, Family/ or exp. Preventive Health Services/
10.	exp Community Health Nursing/ or exp. Community Health Workers/ or exp. Community Health Centers/ or exp. Community Mental Health Services/ or exp. Community Pharmacy Services/ or exp. Community Health Services/
11.	(primary care or primary health care or primary healthcare).af.
12.	(general practice$ or family practice$ or family medicine or family physician$ or medical home$).af.
13.	(community health or community nurs$ or community mental health service$ or community pharmacy service$ or community controlled health).af.
14.	9 or 10 or 11 or 12 or 13
15.	exp Oceanic Ancestry Group/ or exp. American Native Continental Ancestry Group/ or exp. Minority Groups/ or exp. Ethnic Groups/
16.	(indigenous or aborigin$ or maori or pacific island$ or torres strait island$ or native american$ or american indian$ or african american$ or hispanic$ or first nation$ or inuit$ or ethnic minorit$).af.
17.	exp Vulnerable Populations/ or exp. Medically Underserved Area/ or exp. Healthcare Disparities/
18.	(vulnerable or disadvantaged or health$ disparit$).af.
19.	15 or 16 or 17 or 18
20.	exp Chronic Disease/ or exp. Disease Management/ or exp. Self care/
21.	(chronic disease$ or disease management or self care or self-management or selfmanagement).af.
22.	exp Asthma/ or exp. Diabetes Mellitus, Type 1/ or exp. Diabetes Mellitus, Type 2/ or exp. Diabetes Mellitus/ or exp. Pulmonary Disease, Chronic Obstructive/ or exp. Depression/ or exp. Long-Term Synaptic Depression/ or exp. Cortical Spreading Depression/ or exp. Depression, Postpartum/ or exp. Depression, Chemical/ or exp. Mental Health/ or exp. Heart Diseases/ or exp. Heart Failure/
23.	(asthma or diabet$ or chronic pulmonary obstructive disease$ or copd or depression or mental health or cardiovascular disease$ or coronary disease$ or heart disease$ or coronary artery disease$ or heart failure or cardiac failure).af.
24.	(health assessment$ or health check$ or screening).af.
25.	20 or 21 or 22 or 23 or 24
26.	3 and 8 and 14 and 19 and 25

## Abbreviations

ABCD: Audit and Best Practice for Chronic Disease
ABCDE: Audit and Best Practice for Chronic Disease Extension
ACCHS: Aboriginal Community Controlled Health Services
CQI: Continuous quality improvement
Indigenous primary health care services: Aboriginal and Torres Strait Islander primary health care services
PDSA: Plan-Do-Study-Act cycles
SAT: Systems assessment tool
